# Supplementary material for: Age-linked suppression of lipoxin A4 associates with cognitive deficits in mice and humans
Source: Transl Psychiatry. 2022 Oct 10;12:439. doi: 10.1038/s41398-022-02208-1 (PMC9551034; doi:10.1038/s41398-022-02208-1)
Supplement: Supplementary file 1 — Supplemental Material [file 41398_2022_2208_MOESM1_ESM.docx]

Supplemental Information

Age-linked suppression of lipoxin A4 associates with cognitive deficits in mice and humans

Fabricio A. Pamplona^1,2,3^*, Gabriela Vitória^1^, Felipe K. Sudo^1^, Felipe C. Ribeiro^4^, Alinny R. Isaac^4^, Carolina A. Moraes^2^, Mariana G. Chauvet^4^, Pitia Flores Ledur^1^, Karina Karmirian^1,5^, Isis M. Ornelas^1,6^, Luciana M. Leo^2^, Bruna Paulsen^7,8^, Gabriel Coutinho^1^, Claudia Drummond^1,9^, Naima Assunção^1^, Bart Vanderborght^1^, Claudio A. Canetti^10^, Hugo C. Castro-Faria-Neto^2^, Paulo Mattos^1^, Sergio T. Ferreira^1,4,10^, Stevens K. Rehen^1,5^, Fernando A. Bozza^1,2^, Mychael V. Lourenco^4^*, Fernanda Tovar-Moll^1^*

^1^D’Or Institute for Research and Education (IDOR), Rio de Janeiro, Brazil

^2^Laboratory of Immunopharmacology, Oswaldo Cruz Institute, Oswaldo Cruz Foundation (FIOCRUZ), Rio de Janeiro, Brazil ^3^Biosciences Program, Federal University for Latin American Integration, Foz do Iguaçu, Brazil

^4^Institute of Medical Biochemistry Leopoldo de Meis; ^5^Department of Genetics, Institute of Biology; ^9^Department of Speech and Hearing Pathology; ^10^Institute of Biophysics Carlos Chagas Filho, Federal University of Rio de Janeiro, Rio de Janeiro, RJ 21941-902, Brazil

^6^Department of Physiological Sciences, Federal University of Espírito Santo, ES 29040-090, Brazil

^7^Department of Stem Cell and Regenerative Biology, Harvard University, Cambridge, MA, USA.

^8^Stanley Center for Psychiatric Research, Broad Institute of MIT and Harvard, Cambridge, MA, USA.

*Correspondence to: fabriciopamplona@gmail.com (F.A. Pamplona), fernanda.tovarmoll@idor.org (F. Tovar-Moll) or mychael@bioqmed.ufrj.br (M.V. Lourenco).


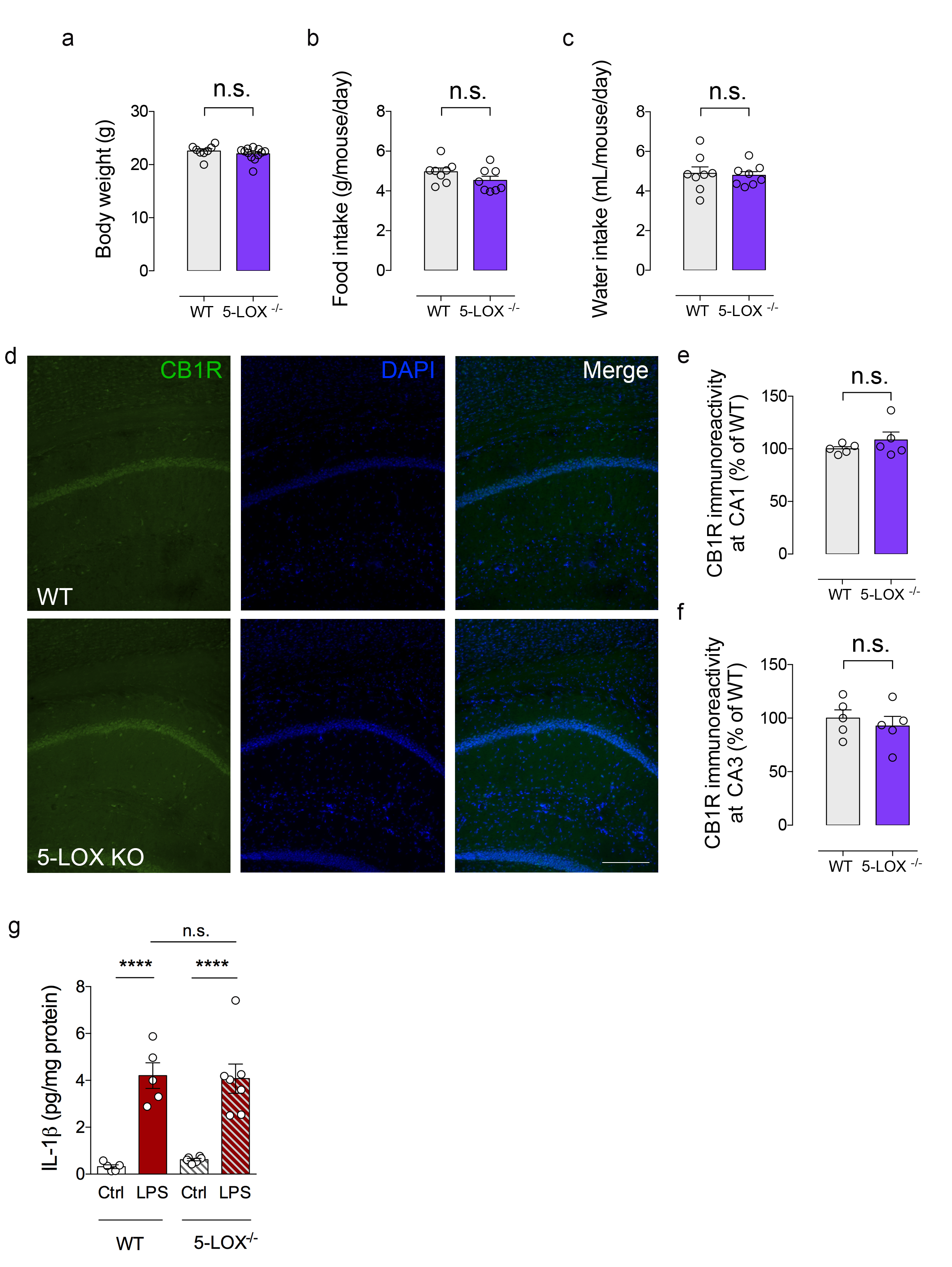


**Supplemental Figure 1. 5-LOX^-/-^ mice do not present alterations in body weight, food/water intake, hippocampal endocannabinoid receptor 1 (CB1R) expression or neuroinflammation-associated IL-1β expression.** (a-c) Body weight (a), 24-hour food intake (b) and 24-hour water intake (c) in adult male 5-LOX^-/-^ or WT mice. (d) Representative images of immunofluorescence experiments (CB1R immunoreactivity: green; DAPI immunoreactivity: blue) in the hippocampal formation of 5-LOX^-/-^ or WT mice. Scale bar: 50 μm. (e,f) Quantification of CB1R immunoreactivity at CA1 (e) and CA3 (f) hippocampal subregions obtained from experiments in *d* (n = 5 mice per group). Unpaired two-tailed Student’s t-test; n.s., non-significant. (g) Brain levels of IL-1β in WT or 5-LOX^-/-^ mice injected with saline or LPS (0.3 mg/kg; i.p.). Two-tailed unpaired two-way ANOVA followed by Holm- Šidák post hoc test; n.s., non-significant; **** p < 0.0001). Graphs show means ± standard error of the mean (SEM). Graphs show mean ± standard error of the mean (SEM).


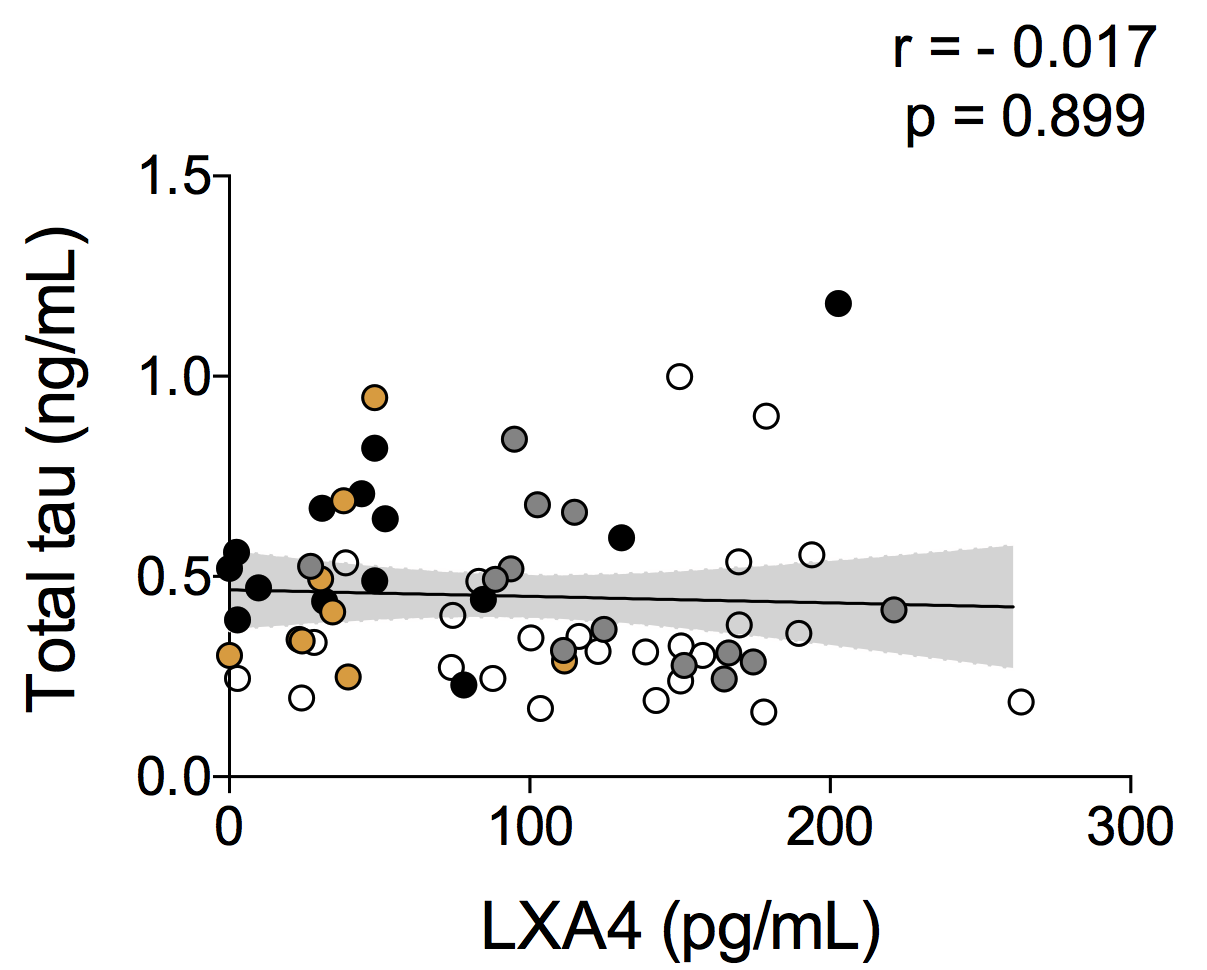


**Supplemental Figure 2. LXA4 does not correlate with total tau in the CSF.** Line represents a partial rank correlation (r and p-values as indicated in graph) and the confidence interval is represented as gray shade. Healthy controls: white symbols; aMCI: grey symbols; AD: black symbols; DLB: golden symbols).
